# Teaching medical students to navigate workplace harassment – preliminary experiences from a pilot workshop in Germany

**DOI:** 10.1186/s12909-025-07853-w

**Published:** 2025-09-10

**Authors:** Sabine Drossard, Iris Warnken

**Affiliations:** 1 Department of General, Visceral, Transplant, Vascular and Pediatric Surgery, University Hospital Würzburg, Oberdürrbacher Str. 6, Würzburg, 97080 Germany; 2 Medical Didactics and Education Research, DEMEDA, Faculty of Medicine, University of Augsburg, Augsburg, Germany

**Keywords:** Sexual harassment, Medical education, Undergraduate education, Workplace violence, Gender discrimination

## Abstract

**Background:**

Sexual harassment in medical education is highly prevalent and can be perpetrated by both faculty and patients. To date, medical students receive no formal training specifically preparing them for handling sexual harassment in the workplace. Additionally, no German-language resources exist that educators can rely on for guidance in dealing with patient-perpetrated sexual harassment.

**Methods:**

A 90-minute practice-oriented workshop on addressing sexual harassment in medical education was developed, implemented, and evaluated at the University of Augsburg. The seminar aims to train medical students in recognizing and responding to workplace harassment. The workshop incorporated interactive methods and role-playing scenarios to facilitate the learning and practice of communication strategies. All participants completed an evaluation questionnaire, providing a retrospective self-assessment of their ability to identify and respond to sexual harassment.

**Results:**

A total of 20 out of 72 students participated in the seminar across four sessions, 20/20 completed the questionnaire. Women reported significantly more personal experiences of sexual harassment while men reported slightly higher tendencies to directly address sexual harassment, but this difference was not statistically significant. Following the workshop, participants reported feeling significantly better prepared in their self-assessment, no significant gender differences were observed. Participants rated the workshop as relevant and practical and strongly endorsed its continuation.

**Conclusion:**

The workshop addresses an existing gap in medical education and serves as a valuable addition to communication training. The use of role-playing exercises and the practice of responses were perceived by participants as highly beneficial. Expanding the program and offering it earlier in the curriculum is recommended.

**Supplementary Information:**

The online version contains supplementary material available at 10.1186/s12909-025-07853-w.

## Introduction

Sexual harassment is highly prevalent in medical education, as demonstrated by various international studies. A 2011 meta-analysis reported that 59% of all medical students worldwide had experienced at least one form of harassment or discrimination, with 33% reporting sexual harassment and 54% experiencing gender-based discrimination [1]. In the United States, a 2018 survey found that 45–50% of all female medical students had been affected by sexual harassment [2]. Recent studies have confirmed similar findings for Germany: At the Medical Faculty of Münster, the Hannover Medical School and Charité Berlin more than half of medical students reported experiencing sexual harassment or gender-based discrimination during their training [3–5]. Perpetrators primarily include patients and senior physicians, among others [3, 6–14].

Several studies have examined the prevalence of sexual harassment in various medical training contexts. In particular, during practical training sessions, situations frequently arise in which patients or teachers intentionally or unintentionally harass or discriminate against trainees [3, 15]. Many of these incidents remain unaddressed [16]. Students frequently struggle to recognize the experienced behavior as harassment [3], while faculty also find it difficult to address misconduct [17].

Although medical education in Germany has increasingly focused on the development of communication skills [18], medical students do not receive targeted training to prepare them for handling sexual harassment in the workplace. There are only limited descriptions of workshops addressing the management of sexual harassment in the medical context, focusing on educators [19] and physicians in postgraduate training [17, 20]. To our knowledge, this is the first description of a workshop targeting medical students.

At the Medical Faculty of the University of Augsburg, which is still in its developmental phase following the enrollment of its first cohort in the winter semester of 2019/2020, medical students have reported experiences of sexual harassment during their training. These reports emerged both within the framework of the mentoring program [21] and through interactions with student representatives. In response, the student representation and the Medical Faculty appointed trusted faculty members in 2022 to address these concerns. Discussions with students highlighted the substantial challenges they face in managing sexual harassment within the context of practical training, partly due to their ongoing uncertainty in their professional roles. Consequently, the Medical Faculty decided to integrate “Workplace Harassment” as a dedicated topic within the longitudinal curriculum on professional identity formation.

## Materials and methods

Based on findings from a qualitative interview study on sexual harassment in medical education (own data, unpublished) and drawing on existing literature on managing sexual harassment in medical settings, a 90-minute seminar on addressing sexual harassment in medical education was developed, implemented, and evaluated at the University of Augsburg. This seminar aimed to train medical students in recognizing and handling sexual harassment during their studies. Its development was carried out in close collaboration with the student representatives. The seminar was integrated into the core curriculum in the ninth semester. Attendance was not mandatory; no assessment was conducted.

### Learning objectives

The following learning objectives were defined: By the end of this seminar, students will be able to….

- Explain the definition of (sexual) harassment.
- Identify and discuss different forms of inappropriate behavior and sexual harassment.
- Recognize and reflect on the importance of personal boundaries.
- Evaluate situations in which personal boundaries are violated and differentiate them from external expectations placed upon individuals.
- Demonstrate sensitivity to others’ boundaries and effectively support individuals experiencing a boundary violation.
- Identify appropriate support services and resources available in case of an incident.
- Describe safe approaches to handling boundary violations and apply communication techniques.

### Structure and methods

The workshop utilized interactive methods and role-playing scenarios to enhance the learning and application of communication strategies. Following an introduction to the definitions and legal framework, students were asked to evaluate various scenarios to heighten their awareness of individual boundaries. Subsequently, they collaborated in small groups to reflect on response strategies, received evidence-based input, and participated in a partner exercise, where they practiced different reactions using sample scenarios to reinforce their skills. It focused on two perpetrator groups: patients and senior physicians. For a detailed overview of the workshop structure and methods, please refer to Appendix 1.

The seminar was conducted jointly by a senior clinician-educator and a medical education researcher, both of whom had relevant professional experience and a shared history of experiencing sexual harassment in clinical settings. Neither of the instructors had an evaluative role with regards to the participating students.

### Evaluation

All participants were asked to complete an evaluation questionnaire in which they provided a retrospective self-assessment of their ability to recognize and respond to sexual harassment. The questionnaire was developed based on a previously described workshop for postgraduate physicians [20] and was adapted to the context of medical students in Augsburg (Appendix 2). To suit the undergraduate population, the questionnaire was modified to reflect the medical student experience. While the original instrument focused exclusively on patient-initiated harassment in clinical environments, in our adaptation professional terminology (e.g., “trainees,” “colleagues”) was replaced with student-appropriate phrasing to include harassment by faculty and supervisors. The harassment response scale was condensed into global competence domains aligned with the workshop’s learning objectives. Previous experiences with sexual harassment and reaction to it were evaluated on a 5-point Likert scale, likelihood of using the trained skills and feedback about the workshop were evaluated on a 5-point Likert scale., data were collected using a 5-point Likert scale both before (pre) and after (post) the intervention. A retrospective pre–post self-assessment format was chosen to reduce response-shift bias [22]. Furthermore, basic demographic data was collected and the participants were given the opportunity to give further comments via two open-ended questions. The adapted tool was reviewed by a student representative for relevance and clarity, though no formal pilot testing was conducted. The evaluation was conducted at the end of the seminar using paper-based questionnaires. Participation in the evaluation was voluntary and anonymous, verbal consent was obtained.

### Statistical analysis

Data were analyzed using Python (Version 3.11). For all continuous variables derived from the retrospective pre–post self-assessment, the distribution of differences between pre- and post-intervention ratings was first tested for normality using the Shapiro-Wilk test. For variables that were normally distributed, paired t-tests were applied to assess changes in self-assessed competence. For non-normally distributed variables, the Wilcoxon signed-rank test was used. Gender-specific differences in pre–post changes were analyzed separately: for normally distributed data using independent samples t-tests, and for non-normally distributed data using the Mann–Whitney U test. All self-assessed competency variables were treated as dependent measures, as each participant provided both pre- and post-intervention assessments. A significance level of α = 0.05 was applied to all tests. Descriptive statistics were used to summarize participant demographics, prior experiences, and workshop feedback.

## Results

The workshop was conducted at the beginning of the winter semester 2023/2024. A total of 20 students attended the seminar across four sessions. Gender distribution of the participants reflected the overall distribution of the study cohort (Table 1). Given a total cohort size of 72 students, the participation rate was 27.8%. Evaluation response rate was 100%.

**Table 1:** Demographics of participants

	Total	Percentage
Gender		
Male	7	35,0%
Female	13	65,0%
Other	0	0,0%
Age		
18–21	0	0,0%
22–25	16	80,0%
26–29	4	20,0%
>30	0	0,0%
Prior experience before medical school		
No	7	35,0%
Yes, academic studies	2	10,0%
Yes, vocational training	4	20,0%
Yes, other	7	35,0%
Previous attendance of training on the topic		
Yes	3	15,0%
No	14	70,0%
Unsure	3	15,0%

Women reported experiencing sexual harassment significantly more frequently than men, while there was no significant difference between men and women regarding having observed sexual harassment. There was no significant difference in reaction to sexual harassment, both as affected person and as bystander (Table 2).

**Table 2:** Previous experiences with and reaction to sexual harassment. 5-point likert scale (1 = never, 5 = very often)

Experiences with sexual harassment	Mean (SD)			*p*-value
	All (*n* = 20)	Male (*n* = 7)	Female (*n* = 13)	
I have experienced sexual harassment personally	2,65 (1,28)	1,71 (0,70)	3,15 (1,23)	0,008
I have reacted to sexual harassment personally	1,90 (1,37)	1,29 (1,67)	2,23 (1,05)	0,081
I have witnessed sexual harassment as a bystander	2,55 (1,02)	2,43 (0,73)	2,62 (1,15)	0,585
I have reacted to sexual harassment as a bystander	1,90 (0,94)	2,00 (1,07)	1,85 (0,86)	0,766

**Fig. 1:**
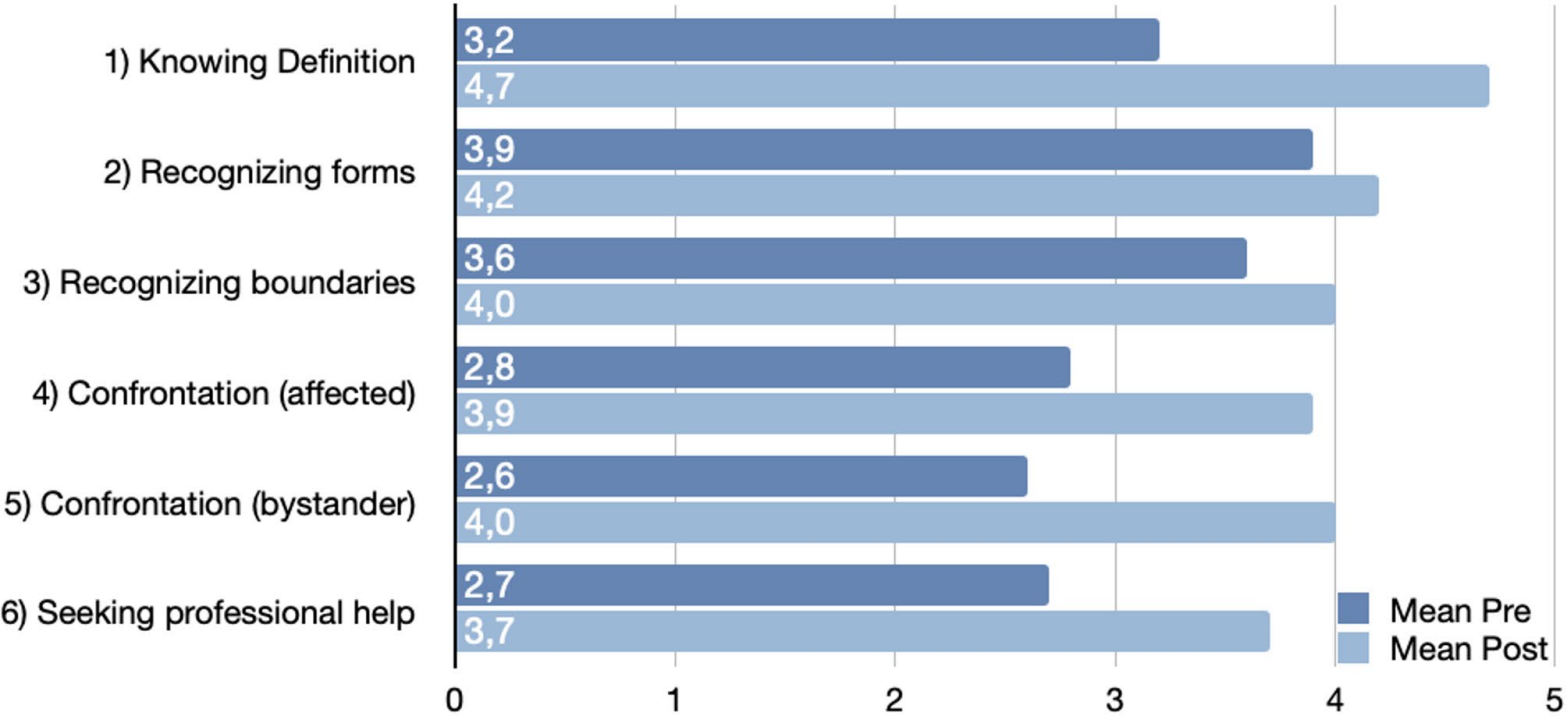
Change in self-evaluation due to intervention: “How would you assess your competency in the following areas?” (Mean, 5-point Likert scale, 1 = very poor, 5 = very good. Pre and post Intervention, *n* = 20)

All students reported an increase in self-assessed competency following the workshop, significant changes were observed for all variables except recognizing forms of harassment (Fig. 1).

The greatest improvements were observed in explaining the definition of sexual harassment (ΔM = 1.45, *p* < 0.001), addressing harassment as a bystander (ΔM = 1.35, *p* < 0.001), and addressing harassment as a victim (ΔM = 1.05, *p* < 0.001). No significant change was found for recognizing forms of harassment (*p* = 0.083). Although mean changes varied between genders, none of these differences reached statistical significance (*p* > 0.05). The largest gender differences were observed in explaining the definition and addressing harassment as a victim (Table 3).

**Table 3:** Change in self-evaluation, mean difference (Δ) and 95% confidence interval. 5-point likert scale (1 = very poor, 5 = very good)

Item		Mean Δ (All, *n* = 20)	95%-CI	*p*-value	Mean Δ (Male, *n* = 7)	Mean Δ (Female, *n* = 13)	*p*-value
1	Knowing Definition	1,45	1,13 − 1,77	< 0,001	1,14	1,62	0,119
2	Recognizing forms	0,30	−0,04 − 0,64	0,083	0,57	0,15	0,241
3	Recognizing boundaries	0,40	0,08 − 0,72	0,021	0,57	0,31	0,327
4	Confrontation (affected)	1,05	0,69 − 1,41	< 0,001	0,71	1,23	0,163
5	Confrontation (bystander)	1,35	0,94 − 1,76	< 0,001	1,43	1,31	0,704
6	Seeking professional help	1,05	0,58 − 1,52	< 0,001	1,43	0,85	0,223

All participants indicated that they considered it likely that they would apply the workshop content and utilize the communication strategies in future situations involving sexual harassment by patients or teachers (Table 4). Male participants (M = 4.29) reported slightly higher likelihoods of directly addressing sexual harassment compared to female participants (M = 3.77); however, this difference was not statistically significant (*p* = 0.075).

**Table 4:** Likelihood of application and feedback on the seminar. Likelihood of application was assessed using a 5-point likert scale (1 = very unlikely, 5 = very likely). feedback on the seminar was measured on a 5-point likert scale (1 = strongly disagree, 5 = strongly agree). M = Mean, SD = StanDard deviation, *n* = 20

**How likely are you to apply one of the communication strategies discussed in the workshop in the future if…**	**M (SD)**
…you experience sexual harassment by patients?	4,3 (0,5)
…you experience sexual harassment by teachers?	3,8 (0,7)
…you witness sexual harassment by patients?	4,2 (0,7)
…you witness sexual harassment by teachers?	3,9 (0,7)
**Feedback**	
The content and examples were relevant to my everyday practice.	4,5 (0,7)
I learned a lot.	4,2 (0,7)
The practical exercises enhanced my understanding and retention of the material.	4,0 (0,9)
I acquired practical skills that I can apply in real-world situations.	4,5 (0,5)
I feel better equipped to handle everyday situations.	4,2 (0,4)
The learning objectives and content were well-selected and effectively aligned.	4,6 (0,5)
I would recommend this workshop to my fellow students.	4,7 (0,6)
Faculty would also benefit from this workshop.	4,9 (0,5)
I found the workshop engaging and enjoyable.	4,6 (0,6)

The student feedback on the workshop was overwhelmingly positive, with all evaluated aspects receiving high ratings. Participants particularly appreciated the relevance of the content to their daily practice (*M* = 4.5, *SD* = 0.7) and the practical applicability of the acquired skills (*M* = 4.5, *SD* = 0.5). The lesson was also well-structured, as indicated by the high rating for the alignment of learning objectives and content (*M* = 4.6, *SD* = 0.5).

Students strongly endorsed the workshop, with a mean rating of 4.7 (SD = 0.6) for recommending it to peers. Interestingly, the highest-rated statement was that faculty members would also benefit from the workshop (*M* = 4.9, *SD* = 0.5), suggesting a perceived need for broader training on the topic. While the practical exercises were seen as beneficial (*M* = 4.0, *SD* = 0.9), they received the lowest score among all evaluated aspects, possibly indicating room for further refinement in their design or implementation. Nonetheless, overall enjoyment of the workshop remained high (*M* = 4.6, *SD* = 0.6) (Table 4).

Open-ended responses indicated that students preferred the workshop to be offered earlier in the curriculum. They expressed a desire for more hands-on training and additional guidance on bystander intervention strategies.

Following the pilot phase, the workshop was integrated into the medical curriculum. Based on student feedback the faculty chose to move the workshop to an earlier point in the curriculum (fifth semester). At the time of manuscript revision, however, no follow-up evaluation data were available.

## Discussion

The positive reception of the pilot workshop highlights the potential value of structured training on handling sexual harassment within medical education. A key strength of this study is that it describes in detail the first evaluated seminar on sexual harassment management for medical students in a German-speaking medical program. A web search indicated that an increasing number of seminars targeting medical students are being offered at German medical schools, highlighting the growing recognition of the relevance of this topic.

The fact that a substantial proportion of students in this study had experienced harassment underscores the urgency of addressing this issue within medical training programs. The findings of this study align with existing research highlighting the high prevalence of sexual harassment in medical education [1, 3–5]. Despite the recognized problem, most medical curricula lack structured training on how to handle sexual harassment in the clinical workplace. While communication skills training has become increasingly integrated into medical education [18, 23], specific guidance and practical exercises on addressing harassment remain absent. Compared to existing workshops that primarily target faculty members or postgraduate trainees [19, 20, 24], this workshop fills an important gap by focusing on medical students, who are often in particularly vulnerable positions during their early clinical experiences [3–5, 10, 12, 25–27].

Involving student representatives in the definition of learning objectives proved effective, highlighting the usefulness of co-creation in the design of targeted interventions [28]. The intervention was designed to be interactive, practical and skills-based, which enabled students to practice real-life scenarios and develop effective response strategies. Role-playing exercises and predefined communication techniques were rated positively by participants, reinforcing previous research that suggests experiential learning approaches are superior to passive instruction in preparing students for challenging interpersonal situations [29, 30]. Furthermore, the evaluation response rate was 100%, indicating strong engagement among participants.

Participants reported feeling better prepared to handle such situations in their professional environment, with statistically significant improvements in multiple self-reported learning outcomes. Notably, no significant gender differences were found in the effectiveness of the training, although female participants reported higher prior exposure to sexual harassment compared to their male counterparts. The variable “recognizing forms of harassment” did not show a statistically significant change, reflecting a possible limitation of the short workshop format, which may not have provided sufficient time or exposure to diverse case examples needed to build pattern recognition and confidence in uncertain contexts. To address this, future iterations of the training could include expanded scenario-based practice and reinforcement sessions over time.

### Limitations

Several limitations must be acknowledged when interpreting the findings of this pilot study. First, participation in the seminar was voluntary, which may have introduced selection bias. Students who already had an interest in the topic or prior experiences with harassment may have been more likely to attend, potentially limiting the generalizability of the results. Additionally, the small sample size (*n* = 20) limits statistical power and precludes robust subgroup analyses. Second, the evaluation relied exclusively on retrospective self-assessment, which—while commonly used in educational research—does not measure actual behavioral change or long-term application of skills. Although retrospective pre–post designs can reduce response-shift bias by allowing learners to better assess their baseline competence post-intervention [22], they are inherently subject to recall bias and social desirability effects [31]. Future studies should incorporate longitudinal follow-up and behavioral measures to assess real-world impact.

### Future implications

The strong student engagement and positive feedback suggest that integrating targeted training on workplace harassment into the medical curriculum may enhance students’ preparedness and confidence. Early implementation could help equip future healthcare professionals with the skills needed to navigate such situations. Notably, students strongly endorsed similar training for faculty, underscoring the broader institutional need for structured education on harassment management. As key role models, faculty play a critical part in shaping the learning environment. Providing them with tools to recognize, address, and prevent harassment may support a cultural shift toward safer, more respectful clinical settings. To enhance effectiveness, repeated training sessions should be implemented, with the initial workshop ideally introduced earlier in the curriculum, preferably before students enter clinical rotations. Based on the pilot experience, we suggest that future adaptations for preclinical learners might include simplified scenarios, increased scaffolding of communication techniques, and greater emphasis on exploring professional roles and boundaries in the early stages of medical education. Making participation mandatory could help ensure that all students receive this essential training. Similar programs should be developed and evaluated for educators and clinical supervisors.

While the current evaluation focused on self-assessed competence immediately after the intervention, future research should explore the long-term impact of such training on actual behavior in clinical settings. Longitudinal studies incorporating follow-up assessments during clinical rotations could help determine whether participants apply the communication strategies in real-world contexts and whether the training influences reporting behavior or professional confidence over time. In addition, the development of objective measures, such as observed structured role-plays or supervisor evaluations, could complement self-report data and provide a more comprehensive understanding of training effects. Expanding the program to other health professions such as nursing or physiotherapy, who are also at risk of harassment, could foster broader team-based awareness and improve coordinated response strategies, thereby increasing the program’s relevance and institutional impact.

## Conclusion

This pilot describes in detail the implementation of a structured, interactive workshop on workplace harassment, reporting evaluation data and exploring the effects on medical students’ self-assessed competence in recognizing and responding to sexual harassment.

The high levels of student engagement and positive feedback indicate that such training is perceived as valuable and relevant by participants. The workshop was positively evaluated by participants, who particularly appreciated role-playing exercises and communication training. Importantly, students strongly endorsed expanding the program and recommended similar training for faculty members. To enhance its impact, the workshop should be offered early in training. While the results suggest perceived improvements in confidence across several learning objectives, these findings are based on retrospective self-report data and must be interpreted with caution. The findings highlight the urgent need for targeted training in medical education, as students currently lack structured guidance on how to handle harassment in clinical settings. Future studies should focus on long-term behavioral outcomes and institutional efforts to create safer learning environments in medical education.

## Supplementary Information

Supplementary Material 1: Appendix 1: Workshop structure (English translation).

Supplementary Material 2: Appendix 2: Questionnaire (English translation).

Supplementary Material 3: Appendix 3: Scenarios for role-play exercise (German and English translation).

Supplementary Material 4: Appendix 4: Questionnaire (German).

## Data Availability

The datasets used and analyzed during the current study are available from the corresponding author on reasonable request.
