# Supplementary material for: Teaching medical students to navigate workplace harassment – preliminary experiences from a pilot workshop in Germany
Source: BMC Med Educ. 2025 Sep 10;25:1251. doi: 10.1186/s12909-025-07853-w (PMC12421763; doi:10.1186/s12909-025-07853-w)
Supplement: Supplementary file 1 — Supplementary Material 1: Appendix 1: Workshop structure (English translation). [file 12909_2025_7853_MOESM1_ESM.pdf]

Workshop Sexual Harassment at the Workplace for Medical Students. Faculty of Medicine, Augsburg University. Sabine Drossard/Iris Warnken

Time	Section	What?	How?	Content	Materials
5	Arrival	Greeting, Learning Objectives, Advance Organizer	Lecture		
10	Definition	Definition<br>Focus of today's seminar<br>„Disclaimer“	Lecture	Definition. Distinction: Sexual Harassment / Sexual Discrimination / Role Expectations<br>"Perpetrator System": Structure that tolerates / covers / normalizes the behavior<br>Focus on harassment. Work with examples – those who wish to speak about personal experiences, please do so after the seminar.	Powerpoint
15	Examples	Evaluating Scenarios<br>Own experiences with harassment	Mentimeter	Raising awareness: It happens. It can happen to anyone. One can talk about it.<br>Recognizing Inappropriate Behavior: Knowing and respecting one's own boundaries<br>Patients vs. Colleagues vs. Leisure<br>Charming ("Compliments") vs. Inappropriate<br>Depends on the situation, the person – listen to your own feelings! (Possibly + inquiry about own experiences)	Mentimeter-Presentation
10	Boundaries	Reflecting on communication strategies	Small groups	Choose an example or own experience and formulate possible behavior	
5	Boundaries	Collecting suggestions	Plenary	Leave as it is	
	Break				
5	Communication Strategies	Presentation and Reflection on Communication Strategies	Lecture	Introduction to "Setting Boundaries" / Evermood clip<br>Role of bystanders: Eye contact vs. conversation afterwards vs. intervention	Presentation Paper / Evermood
10	Communication of Boundaries	Developing a guide	Plenary	What is important? Collecting points<br>3 points: Behavior, Effect, Boundary<br>I set the boundary<br>I don't need arguments<br>I can and must communicate clearly where my boundary is	Whiteboard
15	Practical Exercise	Own example or example on paper	Partner exercise	Draw a paper, partner exercise course. Possible prompts: Stand on chair, sit down, speak loudly, speak softly, eye contact vs. look at the floor, etc.	+ Handout
5	Points of Contact	Presenting points of contact	Lecture	Supervisors, Evermood, Trusted instructors, etc.	+ Handout
10	Reflection / Conclusion	Outlook: Transfer to own experiences<br>Feedback	Mentimeter	Collect impressions, what did I take away today?<br>What’s missing?	Mentimeter-Presentation<br>+ Evaluation
