# Supplementary material for: Teaching medical students to navigate workplace harassment – preliminary experiences from a pilot workshop in Germany
Source: BMC Med Educ. 2025 Sep 10;25:1251. doi: 10.1186/s12909-025-07853-w (PMC12421763; doi:10.1186/s12909-025-07853-w)
Supplement: Supplementary file 2 — Supplementary Material 2: Appendix 2: Questionnaire (English translation). [file 12909_2025_7853_MOESM2_ESM.pdf]

Sentences for Role-Play (Translation)

Patient: "Why don't you come lie down in bed with me?"

Patient: "I think my intimate area needs to be examined as well."

Patient: "I'm being discharged today. Don't you want to go have dinner with me tonight?"

Patient: "I feel better just seeing you. It's a shame I'm not 20 years younger…"

Patient: "You are really the most beautiful one here on the ward!"

Senior Physician: "Feel for yourself! The patient will surely enjoy it if you touch him."

Senior Physician: "It's supposed to rain on Saturday, it will be a perfect cuddling weekend. Or don't you have a boyfriend/girlfriend?"

Senior Physician: "That scrub fits you very well."

Senior Physician: "Where's your pretty colleague?"

Senior Physician: "The students today… They're all prettier than the next!"

Sätze für Rollenspiel

Patient*in: „Legen Sie sich doch mal mit zu mir ins Bett“

Patient*in: „Ich glaube, mein Intimbereich muss auch noch untersucht werden“

Patient*in: „Ich werde ja heute entlassen, wollen Sie nicht heute Abend mit mir essen gehen?“

Patient*in: „Wenn ich Sie sehe, geht es mir gleich besser. Schade, dass ich nicht 20 Jahre jünger bin…“

Patient*in: „Sie sind wirklich der / die Hübscheste hier auf Station!“

OA/OÄ: „Tasten Sie mal! Der Patient freut sich sicher, wenn Sie ihn anfassen.“

OA/OÄ: „Am Samstag solls ja regnen, das wird sicher ein richtiges Kuschelwochenende. Oder haben Sie keinen Freund? / keine Freundin?

OA/OÄ: „Der Kasack sitzt aber sehr knackig bei Ihnen“

OA/OÄ: „Wo ist denn Ihre hübsche Kollegin?“

OA/OÄ: „Mensch, die Studis von heute… Hier ist ja eine hübscher als die andere!“
