# Supplementary material for: Teaching medical students to navigate workplace harassment – preliminary experiences from a pilot workshop in Germany
Source: BMC Med Educ. 2025 Sep 10;25:1251. doi: 10.1186/s12909-025-07853-w (PMC12421763; doi:10.1186/s12909-025-07853-w)
Supplement: Supplementary file 3 — Supplementary Material 3: Appendix 3: Scenarios for role-play exercise (German and English translation). [file 12909_2025_7853_MOESM3_ESM.pdf]

EVALUATION UND FRAGEBOGEN ZUM SEMINAR "SEXUELLE BELÄSTIGUNG AM ARBEITSPLATZ"

WINTERSEMESTER 2023 /UNIVERSITÄT AUGSBURG, MEDIZINISCHE FAKULTÄT

TEIL 1: Statistik

1.1 Wie alt sind Sie?
- ◯ 18-21
- ◯ 22-25
- ◯ 26-29
- ◯ 30 und älter

1.2 Was ist Ihr Geschlecht?
- ◯ weiblich
- ◯ männlich
- ◯ divers
- ◯ Möchte ich nicht angeben

1.3 Haben Sie vor dem Medizinstudium bereits etwas anderes gelernt oder gearbeitet?
- ◯ Nein
- ◯ Ja, anderes Studium
- ◯ Ja, Berufsausbildung
- ◯ Ja, sonstiges (z.B. Praktikum, FSJ)

1.4 Haben Sie vorher schon einmal Workshops oder Seminare zum Thema sexuelle Belästigung am Arbeitsplatz besucht?
- ◯ Ja
- ◯ Nein
- ◯ unsicher
- ◯ Möchte ich nicht angeben

1.5 – 1.8 Welche Erfahrungen haben Sie bisher mit sexueller Belästigung im Medizinstudium gemacht?

		Nie	Selten	Manch-mal	Oft	Sehr oft	Trifft nicht zu
1.5	Ich habe sexuelle Belästigung **als Betroffene*r** erlebt	◯	◯	◯	◯	◯	◯
1.6	Falls ja: ich habe in der Situation **als Betroffene*r** etwas gesagt	◯	◯	◯	◯	◯	◯
1.7	Ich habe sexuelle Belästigung **als Beobachter*in** erlebt	◯	◯	◯	◯	◯	◯
1.8	Falls ja: ich habe in der Situation **als Beobachter*in** etwas gesagt	◯	◯	◯	◯	◯	◯

TEIL 2: Lernziele

2.1 - 2.6 Wie schätzten Sie Ihre Kompetenz in den folgenden Bereichen ein, **BEVOR** Sie am heutigen Seminar teilgenommen hatten?

		Sehr schlecht	Eher schlecht	Weder gut noch schlecht	Eher Gut	Sehr gut
2.1	Die Definition von sexueller Belästigung zu erläutern	◯	◯	◯	◯	◯
2.2	Verschiedene Formen der sexuellen Belästigung am Arbeitsplatz zu erkennen	◯	◯	◯	◯	◯
2.3	Zu beurteilen, wo meine eigenen Grenzen liegen und wahrzunehmen, wenn diese verletzt werden.	◯	◯	◯	◯	◯
2.4	Sexuelle Belästigung direkt anzusprechen, wenn **ich** davon betroffen bin	◯	◯	◯	◯	◯
2.5	Sexuelle Belästigung direkt anzusprechen, wenn **jemand anderes** davon betroffen ist	◯	◯	◯	◯	◯
2.6	Professionelle Hilfe aufzusuchen, wenn ich von sexueller Belästigung betroffen bin	◯	◯	◯	◯	◯

2.7 - 2.12 Wie schätzen Sie Ihre Kompetenz in den folgenden Bereichen ein, **NACHDEM** Sie am heutigen Seminar teilgenommen haben?

		Sehr schlecht	Eher schlecht	Weder gut noch schlecht	Eher Gut	Sehr gut
2.7	Die Definition von sexueller Belästigung zu erläutern	◯	◯	◯	◯	◯
2.8	Verschiedene Formen der sexuellen Belästigung am Arbeitsplatz zu erkennen	◯	◯	◯	◯	◯

Bitte wenden

**2.9**	Zu beurteilen, wo meine eigenen Grenzen liegen und wahrzunehmen, wenn diese verletzt werden.	◯	◯	◯	◯	◯
**2.10**	Sexuelle Belästigung direkt anzusprechen, wenn **ich** davon betroffen bin	◯	◯	◯	◯	◯
**2.11**	Sexuelle Belästigung direkt anzusprechen, wenn **jemand anderes** davon betroffen ist	◯	◯	◯	◯	◯
**2.12**	Professionelle Hilfe aufzusuchen, wenn ich von sexueller Belästigung betroffen bin	◯	◯	◯	◯	◯

2.13 – 2.16 Was denken Sie, wie wahrscheinlich ist es, dass Sie eine der im Seminar besprochenen Kommunikationsstrategien in Zukunft anwenden werden, wenn…. (Wahrs. = wahrscheinlich)

		**Sehr unwahrs.**	**Eher unwahrs.**	**Weder noch**	**Eher wahrs.**	**Sehr wahrs.**
**2.13**	**Sie selbst** von sexueller Belästigung **durch Patient*innen** betroffen sind?	◯	◯	◯	◯	◯
**2.14**	**Sie selbst** von sexueller Belästigung **durch Lehrende** betroffen sind?	◯	◯	◯	◯	◯
**2.15**	Sie sexuelle Belästigung **durch Patient*innen als Beobachter*in** wahrnehmen?	◯	◯	◯	◯	◯
**2.16**	Sie sexuelle Belästigung **durch Lehrende als Beobachter*in** wahrnehmen?	◯	◯	◯	◯	◯

TEIL 3: Feedback

		**Stimme über-haupt nicht zu**	**Stimme eher nicht zu**	**Teils / teils**	**Stimme eher zu**	**Stimme voll-ständig zu**
**3.1**	Die Inhalte und Beispiele waren relevant für meinen Alltag.	◯	◯	◯	◯	◯
**3.2**	Ich habe viel dazugelernt.	◯	◯	◯	◯	◯
**3.3**	Die praktischen Übungen haben mir geholfen, das Gelernte zu vertiefen.	◯	◯	◯	◯	◯
**3.4**	Ich habe etwas gelernt, was ich im Alltag anwenden kann.	◯	◯	◯	◯	◯
**3.5**	Ich fühle mich jetzt besser auf Alltagssituationen vorbereitet.	◯	◯	◯	◯	◯
**3.6**	Die Lernziele und Inhalte waren gut ausgewählt und aufeinander abgestimmt.	◯	◯	◯	◯	◯
**3.7**	Ich würde das Seminar meinen Kommiliton*innen weiterempfehlen.	◯	◯	◯	◯	◯
**3.8**	Lehrende würden ebenfalls von dem Seminar profitieren.	◯	◯	◯	◯	◯
**3.9**	Das Seminar hat mir Spaß gemacht.	◯	◯	◯	◯	◯

3.10 Haben Sie Anregungen oder Vorschläge für die zukünftige Ausgestaltung des Seminars?

____

____

____

3.11 Möchten Sie sonst noch etwas anmerken?

____

____
