# Supplementary material for: Teaching medical students to navigate workplace harassment – preliminary experiences from a pilot workshop in Germany
Source: BMC Med Educ. 2025 Sep 10;25:1251. doi: 10.1186/s12909-025-07853-w (PMC12421763; doi:10.1186/s12909-025-07853-w)
Supplement: Supplementary file 4 — Supplementary Material 4: Appendix 4: Questionnaire (German). [file 12909_2025_7853_MOESM4_ESM.pdf]

EVALUATION AND SURVEY FOR THE WORKSHOP "SEXUAL HARASSMENT IN THE WORKPLACE"

WINTER SEMESTER 2023 / UNIVERSITY OF AUGSBURG, MEDICAL FACULTY

PART 1: Demographics

1.1 What is your age?
- ◯ 18-21
- ◯ 22-25
- ◯ 26-29
- ◯ 30 und älter

1.2 What is your gender?
- ◯ female
- ◯ male
- ◯ other
- ◯ Prefer not to say

1.3 Did you have any prior education or work experience before studying medicine?
- ◯ No
- ◯ Yes, academic studies
- ◯ Yes, vocational training
- ◯ Yes, other

1.4 Have you previously attended any workshops or seminars on sexual harassment in the workplace?
- ◯ Yes
- ◯ No
- ◯ Not sure
- ◯ Prefer not to say

1.5 – 1.8 What experiences have you had with sexual harassment during your medical studies?

		Never	Rarely	Occasio-nally	Often	Very often	Not applicable
1.5	I have **experienced** sexual harassment **personally**.	◯	◯	◯	◯	◯	◯
1.6	If yes: I spoke up about the harassment **as affected person.**	◯	◯	◯	◯	◯	◯
1.7	I have **witnessed** sexual harassment **as a bystander**.	◯	◯	◯	◯	◯	◯
1.8	If yes: I spoke up about the harassment **as a bystander**.	◯	◯	◯	◯	◯	◯

TEIL 2: Learning Objectives

2.1 - 2.6 How would you assess your competency in the following areas **before** participating in today's workshop?

		Very poor	Poor	Neutral	Good	Very good
2.1	Explaining the definition of sexual harassment	◯	◯	◯	◯	◯
2.2	Recognizing different forms of sexual harassment in the workplace	◯	◯	◯	◯	◯
2.3	Identifying personal boundaries and recognizing when they are violated	◯	◯	◯	◯	◯
2.4	Directly addressing sexual harassment when **I** am **personally** affected	◯	◯	◯	◯	◯
2.5	Directly addressing sexual harassment when **someone else** is affected	◯	◯	◯	◯	◯
2.6	Seeking professional help when I am affected by sexual harassment	◯	◯	◯	◯	◯

2.7 - 2.12 How would you assess your competency in the following areas **after** participating in today's workshop?

		Very poor	Poor	Neutral	Good	Very good
2.7	Explaining the definition of sexual harassment	◯	◯	◯	◯	◯
2.8	Recognizing different forms of sexual harassment in the workplace	◯	◯	◯	◯	◯
2.9	Identifying personal boundaries and recognizing when they are violated	◯	◯	◯	◯	◯
2.10	Directly addressing sexual harassment when **I** am **personally** affected	◯	◯	◯	◯	◯
2.11	Directly addressing sexual harassment when **someone else** is affected	◯	◯	◯	◯	◯
2.12	Seeking professional help when I am affected by sexual harassment	◯	◯	◯	◯	◯

Please turn

2.13 – 2.16 How likely are you to apply one of the communication strategies discussed in the workshop in the future if…

		Very unlikely	Unlikely	Neutral	Likely	Very likely
**2.13**	…you **experience** sexual harassment by **patients**?	◯	◯	◯	◯	◯
**2.14**	…you **experience** sexual harassment by **teachers**?	◯	◯	◯	◯	◯
**2.15**	…you **witness** sexual harassment by **patients**?	◯	◯	◯	◯	◯
**2.16**	…you **witness** sexual harassment by **teachers**?	◯	◯	◯	◯	◯

PART 3: Feedback

		Strongly disagree	Disagree	Neutral	Agree	Strongly agree
**3.1**	The content and examples were relevant to my daily practice.	◯	◯	◯	◯	◯
**3.2**	I learned a lot.	◯	◯	◯	◯	◯
**3.3**	The practical exercises enhanced my understanding and retention of the material.	◯	◯	◯	◯	◯
**3.4**	I acquired practical skills that I can apply in real-world situations.	◯	◯	◯	◯	◯
**3.5**	I feel better equipped to handle everyday situations.	◯	◯	◯	◯	◯
**3.6**	The learning objectives and content were well selected and effectively aligned.	◯	◯	◯	◯	◯
**3.7**	I would recommend this seminar to my fellow students.	◯	◯	◯	◯	◯
**3.8**	Faculty would also benefit from this seminar.	◯	◯	◯	◯	◯
**3.9**	I found the workshop engaging and enjoyable.	◯	◯	◯	◯	◯

3.10 Do you have any suggestions for improving the seminar in the future?

____

____

____

3.11 Would you like to add any other comments?

____

____
